# Behavioural and cognitive profiles in frontotemporal dementia and Alzheimer’s disease: a longitudinal study

**DOI:** 10.1007/s00415-025-13025-z

**Published:** 2025-03-21

**Authors:** Xin Zhang, Muireann Irish, Olivier Piguet, Rebekah M. Ahmed

**Affiliations:** 1 Department of Clinical Neurosciences, Memory and Cognition Clinic, Royal Prince Alfred Hospital, Sydney, NSW Australia; 2 Brain and Mind Centre, The University of Sydney, Sydney, NSW Australia; 3 School of Psychology, The University of Sydney, Sydney, NSW Australia; 4 UCL Queen Square Institute of Neurology, University College London, London, United Kingdom

**Keywords:** Dementia, Frontotemporal dementia, Alzheimer’s disease, Behavioural neurology

## Abstract

**Introduction:**

Longitudinal comparative characterisation of dementia syndromes may aid differential diagnosis, prognostication and intervention implementation.

**Methods:**

We compared the behavioural and cognitive characteristics of 84 behavioural variant frontotemporal dementia (bvFTD), 29 left and 14 right-dominant semantic dementia (SDL and SDR) and 49 Alzheimer’s disease (AD) patients over a follow-up period of 2.4 ± 1.6 years using the Cambridge Behavioural Inventory Revised (CBI-R) and Addenbrooke’s Cognitive Examination third edition (ACE-III).

**Results:**

Linear mixed modelling of time effects found progression of all CBI-R domains, aside from sleep, beliefs and mood domains, and all ACE-III domains. Modelling of group effects found that bvFTD had greater symptoms than AD in most CBI-R domains. Notably, SDL and SDR compared differently with AD and bvFTD; whilst SDR did not differ significantly from bvFTD in any CBI-R domain, SDL had less severe symptoms than bvFTD in everyday skills, motivation, sleep and eating habits; whilst SDL had greater disturbances in abnormal behaviour and stereotypic behaviour than AD, SDR had greater disturbances in addition in motivation and eating habits. Motivation, eating habits, abnormal behaviour and stereotypic behaviour were the most frequently different behavioural domains between groups.

**Conclusion:**

We have shown that the combined, longitudinal use of existing behavioural and cognitive assessments could capture distinct clinical profiles of common and rare dementia syndromes. Our findings also highlight the importance of select behavioural domains such as motivation and the usefulness of separate clinical characterisations of SDL and SDR.

**Supplementary Information:**

The online version contains supplementary material available at 10.1007/s00415-025-13025-z.

## Introduction

Frontotemporal dementia (FTD) encompasses a heterogeneous spectrum of diseases that involve degeneration of the brain’s frontal and temporal lobes. Behavioural-variant frontotemporal dementia (bvFTD), semantic dementia (SD; also known as semantic variant of primary progressive aphasia) and progressive nonfluent aphasia (PNFA, also known as nonfluent variant primary progressive aphasia) are the main clinical variants of FTD [1]. BvFTD is the most common variant and is characterised by changes in behaviour, personality, executive and social cognition in the context of prefrontal and insular degeneration. SD is characterised by early and profound loss of semantic knowledge, attributable to progressive degeneration of the anterior temporal lobe (ATL), generally more pronounced on the left than the right hemisphere. In 30% of SD cases, the opposite pattern of brain atrophy is observed, with greater right than left ATL involvement [2, 3]. These patients tend to show early prosopagnosia and behavioural changes, in addition to language disturbances [1, 4–7]. Based on this differentiation, SD has often been divided into left-dominant SD (SDL) and right-dominant SD (SDR) [2, 7, 8].

Importantly, many clinical symptoms overlap across FTD variants. Patients diagnosed with SD exhibit a degree of clinical, imaging and pathological overlap with bvFTD [1]. This is most notable for those diagnosed with SDR who tend to show socioemotional and behavioural disturbances comparable to that seen in bvFTD [9]. Similarly, semantic and language deficits typical in SD are also observed in bvFTD [9]. In addition, some bvFTD patients will experience episodic memory disturbances of a severity similar to that observed in Alzheimer’s disease (AD) [10]. Conversely, a subset of AD patients will exhibit early dysexecutive/behavioural presentations driven by underlying frontal atrophy, further complicating its differentiation from bvFTD [11, 12].

The relative rarity of some FTD variants may reflect the lack of sensitivity of existing cognitive and behavioural screening measures, resulting in bias towards the detection of common dementias such as AD and bvFTD. Several approaches have been proposed to address this bias and to better reflect the diversity within the spectrum of FTD disorders. These approaches range from using combinations of clinical, radiological and pathological features transdiagnostically to identify the hierarchy of key behavioural and cognitive measures [1]. Varying revisions of standard measures of global cognitive function have been recommended to include symptoms more specific for particular FTD variants, such as emotional apathy for bvFTD and prosopagnosia for SDR [7, 13].

The progressive nature of these neurodegenerative brain disorders is associated with evolving clinical features, which tend to converge with disease progression [14]. This increasing overlap reflects the diffuse and progressive nature of the underlying pathological processes in the brain. Most notably, the laterality of the ATL atrophy observed early in SDL and SDR tends to blur and converge with disease progression [15]. Longitudinal studies in SD have demonstrated that irrespective of the predominant side of atrophy at onset, the contralateral ATL inevitably becomes affected [2]. Longitudinal characterisation of these SD syndromes offers an important means of delineating common and unique symptoms, as well as clarifying how these symptoms evolve and converge over time. However, few such studies are available and amongst these many have tended to focus on single domains of cognition in isolation [2, 7, 11, 16, 17].

The relative paucity of longitudinal behavioural studies in FTD likely reflects difficulties in recruiting and retaining patients over long periods given the progressive nature of the disease. The prominent behavioural disturbances in these syndromes further limit the capacity to conduct comprehensive assessments and impact carer engagement. Keeping behavioural assessments consistent over ensuing follow-up periods can be further challenging as cognitive functions become increasingly compromised. In this regard, the Cambridge Behavioural Inventory Revised (CBI-R) has proven a useful clinical tool. As an informant-based quantitative questionnaire, it efficiently captures the diverse behavioural manifestations of dementia syndromes and has demonstrated clinical utility in discriminating bvFTD from AD [18]. Similarly, the Addenbrooke’s Cognitive Examination (ACE-III) is a validated measure of cognitive function, widely used in the clinical assessment and follow-up of FTD populations [19, 20]. Both measures are easy and quick to administer and, in the case of the ACE-III, generally well tolerated over time [21]. As such, these instruments are well-suited for repeated assessments in clinical settings. Despite these advantages, comprehensive large-scale longitudinal investigations of the cognitive and behavioural profiles across the FTD spectrum using these tools remain relatively scarce [2, 7, 17, 22].

Therefore, the objectives of this study were twofold. First, we sought to explore the longitudinal trajectories of behavioural and cognitive profiles in bvFTD, SDL, SDR, and typical amnestic AD using the CBI-R and ACE-III. Second, we sought to identify common and unique profiles that may improve the differentiation across these clinical groups.

## Methods

### Participants

Participants with the diagnoses of typical amnestic AD, probable bvFTD and SD were recruited between 2010 and 2019 from FRONTIER, the Frontotemporal Dementia research group in Sydney, Australia. Diagnoses were established according to current consensus diagnostic criteria by a multidisciplinary team comprising a senior neurologist, a clinical neuropsychologist, and an occupational therapist [23–25]. As part of the diagnostic process, all participants underwent comprehensive clinical evaluation (including history and examination), neuropsychological assessment (neuropsychological battery additional to the ACE-III), and structural brain MRI. SD was further classified as SDL or SDR based on the predominant side of anterior temporal lobe atrophy on MRI at entry into the study. Exclusion criteria for participants included a prior history of primary neurological, or psychiatric disorders, alcohol and other drug misuse, traumatic brain injury, and limited English proficiency. Disease duration was defined as years elapsed between the onset of symptoms and baseline assessment.

All participants completed at least one longitudinal follow-up, during which clinical and neuropsychological assessments were repeated. The target time interval between baseline and follow-up neuropsychological assessments was 3 years. Previous longitudinal studies have shown the progression of clinical features within this period [17, 26]. In practise, this time interval varied due to logistical limitations and clinical necessity.

### Behavioural and cognitive measures

All participants underwent a comprehensive assessment to determine disease severity, cognitive functioning, presence of behavioural, mood and psychiatric symptoms, as well as caregiver burden at both baseline and follow-up. Disease severity was established using the clinician-administered Clinical Dementia Rating Sum of Boxes (CDR SOB) and Clinical Dementia Rating-Frontotemporal Lobar Degeneration Sum of Boxes (CDR-FTLD SOB). The CDR is a widely used instrument to rate dementia severity in AD based on functional abilities [27]. The CDR-FTLD is an extended version of the CDR that was developed to include additional domains for language and behaviour, designed to be inclusive of both FTD and AD [28].

Cognitive functioning was determined using the Addenbrooke’s Cognitive Examination, either the Revised (ACE-R) or the third edition (ACE-III) [29]. The ACE is a cognitive screening instrument that measures the integrity of five cognitive domains: attention, memory, verbal fluency, language, and visuospatial abilities. ACE-R scores were converted into ACE-III scores in line with previously published algorithms [20].

The behavioural and psychiatric symptoms of participants were rated by their carers using the Cambridge Behavioural Inventory Revised (CBI-R) [18]. This 45-item questionnaire is divided into ten subsections (memory and orientation, everyday skills, self-care, mood, abnormal behaviour, beliefs, eating habits, sleep, stereotypic behaviours, and motivation). Ratings per question range on a scale of 0 to 4 with higher scores indicating elevated frequency and/or severity of behavioural and psychiatric symptoms.

### Statistical analyses

Analyses were conducted using IBM SPSS statistics (Version 26.0). Kolmogorov–Smirnov tests were used to assess suitability for parametric analysis. Comparisons of demographical and clinical variables were conducted using parametric t-tests or nonparametric Kruskal–Wallis H tests. Linear mixed effects modelling was used to examine the CBI-R and ACE-III data across diagnostic groups and time. The mixed effect models enabled us to account for variability in individual patient CBI-R or ACE-III scores at baseline and the heterogeneity of follow-up times. Fixed effects in the model included time (baseline and follow-up), diagnostic group (AD, bvFTD, SDL, SDR) and the interaction between time and diagnostic group. The only random effect included was individual patient CBI-R or ACE-III variability at baseline. Statistical significance in pairwise comparisons of fixed effects was set at *p* < 0.05 following Bonferroni correction. The estimated marginal means from each model were plotted and grouped according to the CBI-R or ACE-III domain.

## Results

### Demographics

A total of 176 participants satisfied the inclusion criteria. Participants consisted of 49 typical ‘amnestic’ AD patients, 84 probable bvFTD patients, and 43 SD patients, of whom 29 were classified as SDL and 14 as SDR. No significant differences were found between diagnostic groups in terms of sex distribution, years of education, age at symptom onset, age at baseline assessment, or time between assessments (*p* value > 0.1) (Table 1). Sixty-seven % of participants from the AD group reported symptom onset before 65 years of age. At baseline, the SDL group had a significantly longer disease duration than the AD group. The bvFTD group had greater functional impairment than the AD group at baseline and follow-up according to CDR SOB and CDR-FTLD SOB. The average follow-up time for all participants was 2.4 ± 1.6 years.

**Table 1:** Demographical and clinical characteristics of the study samples

	AD	bvFTD	SDL	SDR	Test Statistic^a^	*p* value	Post hoc^b^
Sample size	49	84	29	14	–	–	–
Sex [M: F]	31:18	56:28	18:11	8:6	0.598	0.897	–
Education [years]	12.4 (3.6)	11.8 (2.7)	12.6 (3.2)	11.9 (3.8)	1.205	0.752	–
Age at symptom onset [years]	61.3 (8.0)	57.4 (8.8)	58.1 (6.9)	59.1 (7.5)	5.776	0.123	–
Age at baseline [years]	64.8 (8.4)	61.6 (9.2)	63.1 (6.7)	63.6 (7.2)	3.395	0.335	–
Disease duration at baseline [years]	3.5 (2.7)	4.3 (3.1)	6.9 (10.5)	4.6 (1.9)	13.021	0.003	AD < SDL**
Time between assessments [years]	2.1 (1.1)	2.5 (1.9)	2.5 (1.5)	2.8 (1.5)	2.588	0.460	–
CDR SOB score							
Baseline	4.1 (1.9)	6.3 (4.2)	4.4 (2.7)	5.2 (3.3)	9.184	< 0.027	AD < bvFTD**
Follow-up	6.4 (3.3)	9.8 (5.2)	7.6 (4.3)	7.3 (3.9)	16.981	< 0.001	AD < bvFTD**
CDR-FTLD SOB score							
Baseline	5.0 (2.3)	8.5 (13.3)	6.8 (3.8)	7.8 (4.4)	16.981	< 0.001	AD < bvFTD**
Follow-up	8.2 (4.3)	13.3 (6.7)	10.9 (5.7)	9.9 (5.3)	21.335	< 0.001	AD < bvFTD**

Data presented denote mean values with standard deviations in parentheses. Unit of measurement presented in square brackets where appropriate

*AD* Alzheimer’s disease, *bvFTD* behavioural variant frontotemporal dementia, *FRS* frontotemporal dementia rating scale, *SDL* left predominant semantic dementia, *SDR* right predominant semantic dementia

^a^Kruskal–Wallis tests were used for all demographical variables except for sex distribution, which was explored using a Chi-Square test

^b^post-hoc tests

^**^denotes statistically significant at an adjusted *p* value threshold of 0.01

### Behavioural measures

**Fig. 1:**
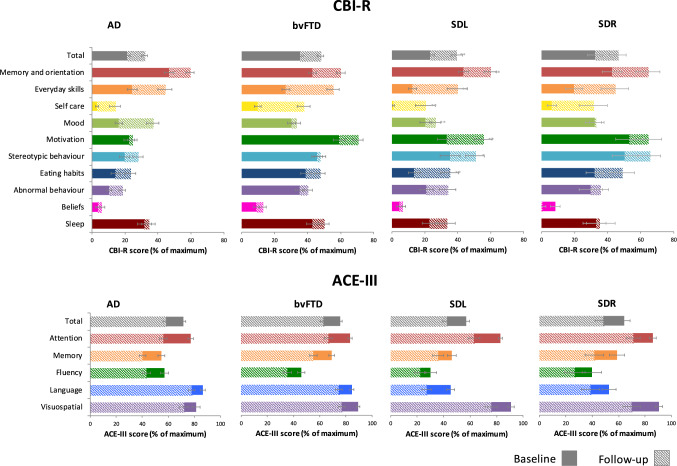
Standardised behavioural and cognitive measures. Mean CBI-R (top panel) and ACE-III subscale (bottom panel) scores as percentages of the maximum score at baseline (solid colour) and follow-up (shaded), with standard error bars. *ACE*-III Addenbrooke’s Cognitive Examination, *AD* Alzheimer’s disease, *bvFTD* behavioural variant frontotemporal dementia, *CBI* Cambridge Behavioural Inventory, *SDL* left predominant semantic dementia, *SDR* right predominant semantic dementia

**Fig. 2:**
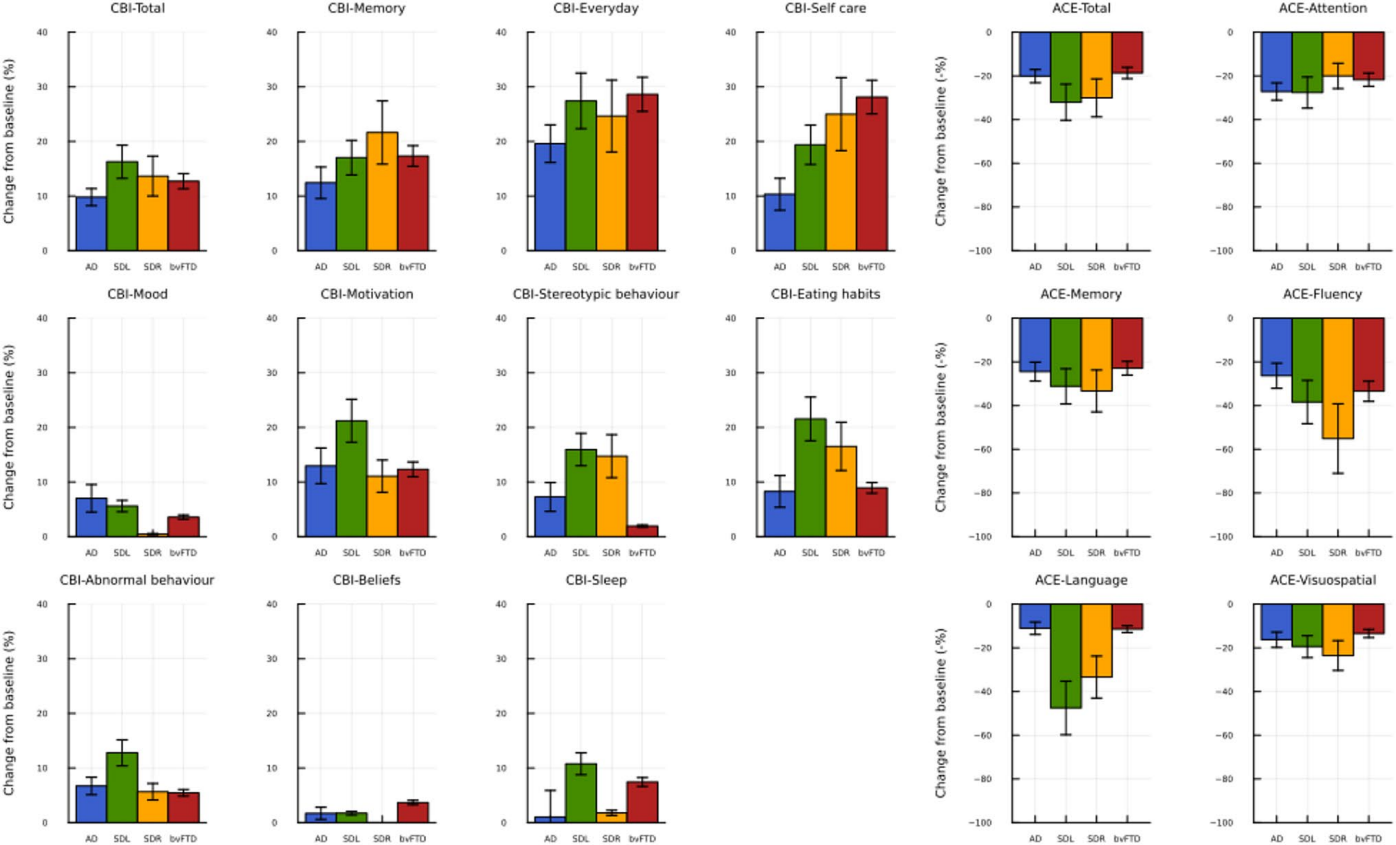
Change in behavioural and cognitive measures. Mean change in CBI-R (left) and ACE-III (right) domain scores between baseline and follow-up. Change is expressed as percentages of the total domain score, with standard error bars. *ACE*-III Addenbrooke’s Cognitive Examination third edition, *AD* Alzheimer’s disease, *bvFTD* behavioural variant frontotemporal dementia, *CBI*-*R* Cambridge Behavioural Inventory Revised, *SDL* left predominant semantic dementia, *SDR* right predominant semantic dementia

Figure 1 and Supplementary Table 1 present the mean CBI-R domain scores as percentages of the maximum score for each diagnostic group at baseline and follow-up. Figure 2 presents the mean change in CBI-R domain scores between follow-up and baseline as percentages of the maximum domain score. By magnitude, the domains with the greatest change were everyday skills and self-care, and the domains with the least change were beliefs, mood and sleep. SDL demonstrated the greatest change by magnitude in total CBI-R score, followed by SDR, bvFTD and lastly AD. Non-parametric testing (Supplementary Table 2) found a significant increase in the self-care domain for the bvFTD compared to the AD group.

Mixed effects modelling revealed a significant time effect with worsening in all CBI-R domains over time, except mood, beliefs and sleep (Table 2). A significant group effect was also present for all domains, except for memory and orientation. These main group effects denoted significantly greater symptom severity in bvFTD than in AD across most CBI-R domains except everyday skills, memory, and self-care. Further, whilst SDR did not differ significantly from bvFTD in any CBI-R domain, SDL had less severe symptoms than bvFTD in everyday skills, motivation, sleep and eating habits. Compared to AD, SDR had greater symptoms in abnormal behaviour, stereotypic behaviour, motivation and eating habits, whilst SDL had greater symptoms only in abnormal behaviour and stereotypic behaviour. SDL and SDR did not differ significantly in any CBI-R domain. Finally, a significant group-by-time interaction was observed exclusively for CBI-R self-care, where post-hoc tests showed a significant increase in bvFTD compared with SDL and AD over time, but not compared with SDR.

**Table 2:** Linear mixed effects modelling of time, group effects and their interaction on behavioural and cognitive measures

	−2 log likelihood/df	F intercept	F time	F group	F group*time	Significant pairwise comparisons Bonferroni corrected
**CBI-R**						
Total	2861.556/11	722.160***	73.055***	14.860***	1.181	bvFTD > AD, SDR > AD, bvFTD > SDL
Memory and orientation	3076.756/11	929.968***	63.939***	0.093	0.614	–
Everyday skills	3276.639/11	261.027***	68.047***	3.723*	0.961	bvFTD > SDL
Self care	3209.317/11	68.804***	54.047***	7.905***	3.935**	bvFTD > AD, bvFTD > SDL
Mood	3067.172/11	277.475***	2.586	6.294***	0.787	bvFTD > AD
Beliefs	2877.932/11	36.163***	1.025	2.873*	0.344	bvFTD > AD
Abnormal behaviour	3094.715/11	248.044***	10.516**	17.272***	1.101	bvFTD > AD, SDL > AD, SDR > AD
Eating habits	3208.979/11	234.806***	31.015***	15.586***	2.131	bvFTD > AD, SDR > AD, bvFTD > SDL
Sleep	3332.780/11	245.386***	3.057	6.098**	0.650	bvFTD > AD, bvFTD > SDL
Stereotypic and motor behaviour	3282.512/11	363.248***	9.888**	12.918***	1.759	bvFTD > AD, SDL > AD, SDR > AD
Motivation	3299.297/11	466.983***	21.811***	24.645***	0.923	bvFTD > AD, SDR > AD, bvFTD > SDL
**ACE-III**						
Total	2471.478/11	1828.439***	80.477***	13.389***	0.338	SDL < AD, SDL < bvFTD, SDR < bvFTD
Attention	2550.366/11	2584.003***	69.965***	3.744*	0.435	AD < bvFTD
Memory	2612.272/11	762.848***	53.302***	11.195***	0.255	AD < bvFTD, SDL < bvFTD
Fluency	2672.204/11	272.516***	44.363***	6.948***	0.439	SDL < AD, SDL < bvFTD
Language	2496.963/11	1750.372***	59.588***	76.518***	2.116	SDL < AD, SDR < AD, SDL < bvFTD, SDR < bvFTD
Visuospatial	2569.826/11	2502.274***	54.029***	1.913	1.149	-

*ACE*-III Addenbrooke’s Cognitive Examination third edition, *AD* Alzheimer’s disease, *bvFTD* behavioural variant frontotemporal dementia, *CBI*-R Cambridge Behavioural Inventory Revised, *SDL* left predominant semantic dementia, *SDR* right predominant semantic dementia

*denotes statistical significance at a *p* value threshold of 0.05;

**denotes statistical significance at a *p* value threshold of 0.01;

***denotes statistical significance at a *p* value threshold of 0.001

**Fig. 3:**
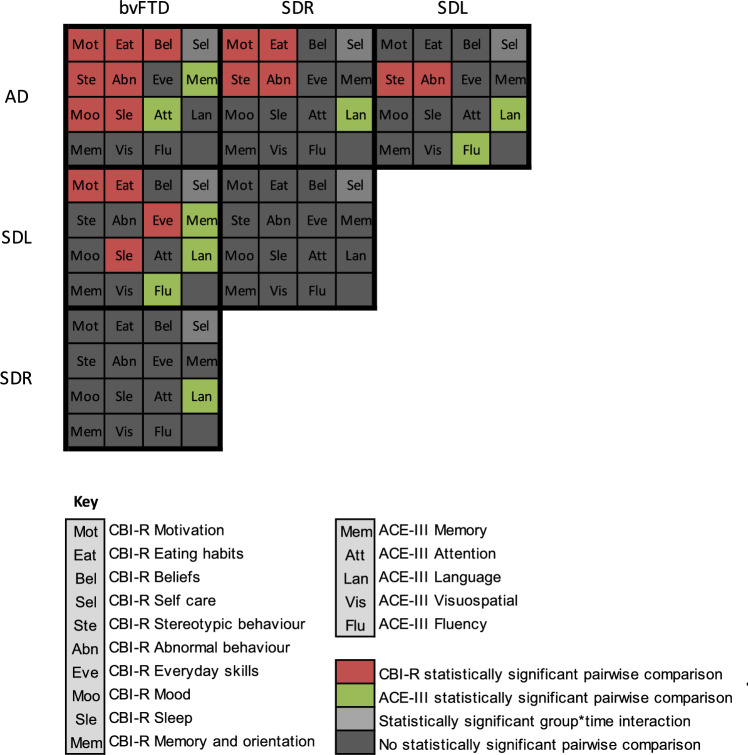
Visualisation of statistically significant pairwise comparisons from linear-mixed effects modelling of group effects. Comparisons satisfying Bonferroni corrected *p* value threshold are highlighted in red (CBI-R) or green (ACE-III). *ACE*-III Addenbrooke’s cognitive examination third edition, *AD* Alzheimer’s disease, *bvFTD* behavioural variant frontotemporal dementia, *CBI*-*R* Cambridge Behavioural Inventory Revised, *SDL* left predominant semantic dementia, *SDR* right predominant semantic dementia

**Fig. 4:**
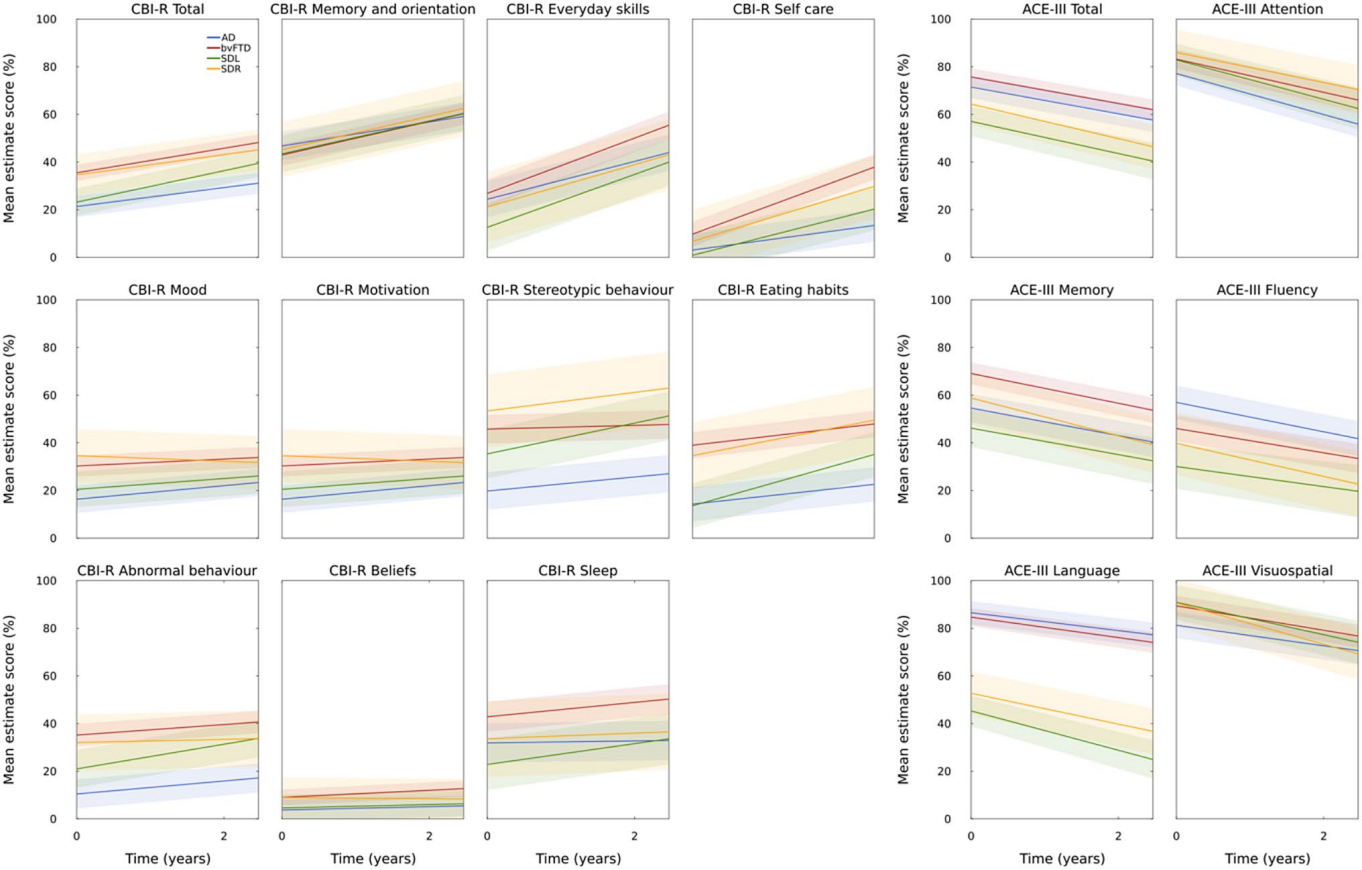
Estimated marginal means of CBI-R and ACE-III scores from linear-mixed effects model. *ACE*-III Addenbrooke’s Cognitive Examination third edition, *AD* Alzheimer’s disease, *bvFTD* behavioural variant frontotemporal dementia, *CBI*-*R* Cambridge Behavioural Inventory Revised, *SDL* left predominant semantic dementia, *SDR* right predominant semantic dementia

The statistically significant pairwise comparisons from the mixed effects modelling are illustrated in Fig. 3 and the estimated marginal means and confidence interval of CBI-R domains from mixed effects modelling are depicted in Fig. 4.

### Cognitive measures

Non-parametric testing found significantly greater declines in the ACE-III language domain for SDL compared to AD or bvFTD, and for SDR compared to AD. (Supplementary Table 2) Mixed effects modelling revealed significant main effects of groups and time for all domains, but no interaction was found (Table 2). Additional post-hoc analyses indicated that AD had greater impairment than bvFTD in attention and memory ACE-III domains. SDR had greater impairment than AD and bvFTD in the language domain. SDL had greater impairment than AD in language and fluency; and than bvFTD in language, fluency and memory. No significant differences were present between SDL and SDR in any ACE-III domain.

## Discussion

Our study demonstrated significant behavioural decline across most domains for all groups with time, cautioning against the interpretation that bvFTD is the only ‘behavioural’ syndrome. We found that distinct profiles of behavioural and cognitive change could be detected when existing clinical measures (the CBI-R and ACE-III) are used in combination longitudinally. The most frequent behavioural differences across groups involved the domains of motivation, eating habits, and abnormal and stereotypic behaviour, features that could be exploited to improve dementia diagnosis and inform the implementation of interventions.

Considering first the behavioural profiles, we found evidence of marked behavioural changes across all dementia groups with disease progression. In particular, the behavioural change in the self-care domain was significantly greater for bvFTD than AD. Importantly, SDL had the greatest increase in total CBI-R by magnitude compared to SDR, bvFTD and AD. Given that SDL has typically been conceptualised as a disorder of language, the anticipation of behavioural exacerbations will help prepare patients and carers.

We found unique profiles of behavioural change with disease progression across the different dementia groups. Echoing existing literature, bvFTD had greater symptoms than AD in most CBI-R domains, except in memory and everyday skills [30–33]. The lack of difference in the CBI-R memory domain may reflect the challenges of asking carers to rate patients on cognitive ability, as greater memory deficits were detected in AD relative to bvFTD using the ACE-III. We further speculate that the lack of a significant difference in the everyday skills domain may reflect the complex and non-specific natures of the abilities queried (e.g. “difficulties using electrical appliances” or “problems handling money”). Several contributing factors may produce the same behavioural symptom. For example, motivational disturbances in bvFTD may result in everyday skills impairment to a similar degree as in AD [32]. Although SDL and SDR had no significant group effects difference on the CBI-R, they followed different profiles when compared with AD and bvFTD. In keeping with previous studies, [9] SDR did not differ significantly from bvFTD in any domain, however, SDL showed milder everyday skills, motivation, sleep and eating habits symptoms relative to bvFTD. Compared with AD, SDL had greater abnormal and stereotypic behaviour, whereas SDR showed additional changes in motivation and eating habits. For the total, motivation and eating habits domains, SDL’s estimated marginal means trajectory initially approximated AD at baseline but later converged with that of bvFTD and SDR. This is consistent with reports of convergence of the clinical profiles of SDL and SDR after approximately 3 years [15]. Whilst no neuroimaging results were included in this study, we tentatively suggest that increased behavioural changes in SDL likely reflect the encroaching of pathology into the right temporal lobe in keeping with previous studies [2, 7, 9, 10, 26, 34].

In terms of behavioural symptoms, the CBI-R domains of motivation, eating habits, abnormal behaviour and stereotypic behaviour appear to be most helpful for the discrimination of different dementia syndromes. Examining motivation for the phenotyping of dementia syndromes is gaining interest in literature [35–38]. Apathy, which is closely related to the motivation domain has been previously shown by some studies to be different for bvFTD compared to other dementias [35, 39]. Whilst eating habits have been traditionally associated with bvFTD [30], our study shows that these changes are also prominent in SDR patients and can help differentiate SDR from AD, highlighting the need to study atrophy patterns and reward pathways associated with eating changes between syndromes [30]. Abnormal and stereotypic behaviour may be useful for differentiating FTD from AD, as they were more severe in all three FTD variants studied, supporting recent calls for the investigation of behavioural rigidity in FTD [40, 41]. The beliefs domain appeared less useful, possibly due to informant underreporting or the lack of hallucinations and delusions in sporadic FTD. Lastly, our finding of self-care impairments progressing faster in bvFTD than in AD and SDL may be useful for prognostication and identification of therapeutic targets.

Dynamic differences between the diagnostic groups were also seen in cognition. As expected, all ACE-III domains significantly declined by follow-up. The SD groups generally had greater language and fluency decline than the non-SD groups. Specifically, SDL had significantly greater declines in the language domain than bvFTD and AD, and SDR had greater language decline than AD. No significant cognitive differences were found between SDL and SDR, suggesting the need for more fine-grained assessments of specific language and cognitive features that typify these syndromes (e.g. prosopagnosia in SDR). However, the ACE-III fluency domain was found to differentiate between SDL from bvFTD and AD, but not for SDR, supportive of phenotypical differences between SDL and SDR. Importantly, we showed that bvFTD had better memory performance than AD, whereas previous longitudinal studies with smaller sample sizes reported a non-significant trend [11]. The better memory performance in bvFTD than SDL also illustrates the cognitive difference between these two FTD variants, however, it is important to note that semantic confounds of these tasks likely drive poor performance in SD [42].

The complementary natures of the CBI-R and ACE-III for capturing differences across dementia subtypes emphasise the importance of a blended approach of using standardised cognitive assessment alongside informant behavioural reports. For example, whilst it is typically difficult to differentiate SDR from bvFTD based on CBI-R alone, the addition of the ACE-III language domain could improve this diagnostic conundrum. Furthermore, the lack of a difference between AD and bvFTD patients on the CBI-R memory domain in the context of distinct differences in the ACE-III memory domain suggests that formal examination of memory is more informative than carer reports in this area.

Our findings add to the growing literature calling for updates of the existing diagnostic criteria for FTD to better delineate variants and address their symptomatic overlap [5, 7, 43]. The constellation of behavioural symptoms in SDR and emerging later in SDL in our study supports the re-conceptualisation of these disorders as more than disorders of language [44–46]. Together with recent proposals for separate criteria for SDR emphasising behavioural changes [7], and emerging reports of SDL and SDR having different molecular pathologies [47], our findings support separate characterizations of SDL and SDR. This may reduce the misdiagnosis of the rarer SDs, an issue exemplified by the inability of the CBI-R to discriminate between bvFTD and SDR in this study [9]. Future criteria and behavioural surveys will need to target what is specifically different between bvFTD and SDR, rather than what is different between bvFTD and all SDs. For example, to better identify SDR, the CBI-R motivation domain may need to be divided to capture the multidimensional components of apathy and anhedonia [35].

Several methodological considerations warrant discussion. The diagnostic process was not blinded to the ACE-III and CBI-R and could not have been blinded to the behavioural and cognitive features reflected by these screening measures. This raises concerns that the independent variable (dementia syndrome) and dependent variables (ACE-III and CBI-R) could be, at least in part, reciprocally related at baseline. However, this concern is mitigated by a substantial non-overlap between ACE-III and CBI-R results and the diagnostic process, which is based on a combination of history, examination and supporting investigations (including neuropsychology assessment and MRI) by a multidisciplinary team according to standard clinical criteria. Information from the ACE-III and CBI-R was of minor importance and not necessarily consulted for the diagnostic process in our tertiary memory service. Nevertheless, given the concerns, we have refrained from interpreting baseline ACE-III and CBI-R differences between dementia syndromes in isolation and directed the focus on longitudinal monitoring of behavioural and cognitive features and how the combined baseline and follow-up scores for the dementia syndromes compare via linear-mixed analysis for group effects. A limitation of this study was the lack of a PNFA group, the inclusion of which would have provided a more comprehensive characterisation of FTD. Nevertheless, data from the groups included were able to demonstrate the usefulness of longitudinal CBI-R and ACE-III assessments. Future studies should consider the incorporation of more disease groups such as PNFA. The rarity of SD and the resultant relatively small sample sizes of SDL and SDR reduced our statistical power to detect significant differences between these two variants. The inclusion of longitudinal imaging and proteinopathy results would help uncover whether the different behavioural progression of the FTD variants are due to molecular nexopathies or progressive neural network degeneration with implications for the delivery of targeted therapeutics. Genetic mutation analyses would have also been helpful for the characterisation of patient groups. Lastly, we could not control for baseline disease duration and disease severity across the dementia syndromes. The finding of SDL having a significantly longer disease duration than AD may be due to several reasons; the better-known amnestic syndrome in AD may have triggered medical consultations earlier, whereas patients with SDL may have presented later as their focal language deficits were easier to mask than the deficits in AD. The greater baseline disease severity for bvFTD compared to AD in the absence of a significant difference in disease duration could reflect earlier impairment of function and behavioural domains that affect function in bvFTD.

In conclusion, our findings underscore the importance of longitudinal assessments of behavioural and cognitive changes across dementia syndromes. The CBI-R appears sensitive to longitudinal behavioural changes and has the potential for monitoring disease progression and treatment response. Complementary use of the CBI-R and ACE-III, and careful interpretation, suggest distinct trajectories for each dementia syndrome that might aid clinical decision-making. With the emergence of disease-modifying therapies for AD and clinical trials for other dementias, there is an urgent need for effective, early detection of dementia symptoms. Standardised screening behavioural and cognitive measures, which are cost-effective and widely accessible at the community level can be as important as the development of blood-based and imaging biomarkers for directing patients to further investigations and treatment. These screening measures can be optimised for more specificity for different dementia syndromes, common and rare alike.

## Supplementary Information

Below is the link to the electronic supplementary material.

Supplementary file1 (DOCX 23 KB)

## Data Availability

Anonymized data not published within this article will be made available by request from any qualified investigator.
