# Supplementary material for: Behavioural and cognitive profiles in frontotemporal dementia and Alzheimer’s disease: a longitudinal study
Source: J Neurol. 2025 Mar 21;272(4):279. doi: 10.1007/s00415-025-13025-z (PMC11928428; doi:10.1007/s00415-025-13025-z)
Supplement: Supplementary file 1 — Supplementary file1 (DOCX 23 KB) [file 415_2025_13025_MOESM1_ESM.docx]

**Supplementary table 1.** Mean CBI-R and ACE-III scores as percentages of the maximum score at baseline and follow-up

|  | AD |  | bvFTD |  | SDL |  | SDR |  |
| --- | --- | --- | --- | --- | --- | --- | --- | --- |
|  | Baseline | Follow-up | Baseline | Follow-up | Baseline | Follow-up | Baseline | Follow-up |
| **CBI-R** |  |  |  |  |  |  |  |  |
| Sample size | 49 | 49 | 84 | 84 | 29 | 29 | 14 | 14 |
| Total | 21.3 (12.2) | 31.8 (12.4) | 35.5 (16.5) | 47.8 (18.2) | 23.2 (14.3) | 39.3 (18.7) | 32.4 (16.8) | 46.1 (19.1) |
| Memory and orientation | 46.8 (20.6) | 59.4 (17.3) | 43.0 (21.5) | 60.1 (24.7) | 43.4 (16.0) | 59.8 (20.9) | 42.6 (22.1) | 64.3 (27.3) |
| Everyday skills | 24.4 (21.1) | 44.2 (29.6) | 26.9 (22.2) | 55.4 (34.0) | 12.6 (13.3) | 39.7 (33.0) | 19.6 (19.6) | 44.3 (31.4) |
| Self-care | 3.0 (6.1) | 13.9 (22.5) | 9.8 (18.8) | 37.6 (35.8) | 0.9 (3.2) | 20.3 (32.6) | 6.3 (10.4) | 31.3 (31.9) |
| Mood | 16.3 (16.1) | 24.5 (19.8) | 30.3 (22.2) | 33.1 (23.9) | 20.5 (18.5) | 26.3 (18.3) | 32.1 (21.4) | 32.6 (13.7) |
| Beliefs | 3.7 (11.3) | 5.4 (16.1) | 9.0 (15.9) | 12.7 (20.6) | 4.6 (9.6) | 6.3 (10.6) | 8.3 (10.8) | 7.7 (12.0) |
| Abnormal behaviour | 10.5 (10.5) | 18.3 (13.7) | 35.2 (24.1) | 40.0 (26.3) | 21.0 (22.2) | 33.8 (27.1) | 29.8 (26.1) | 35.4 (19.2) |
| Eating habits | 14.3 (19.2) | 23.1 (23.8) | 39.0 (28.7) | 47.5 (28.5) | 13.6 (19.4) | 34.9 (29.4) | 32.1 (19.6) | 48.7 (28.2) |
| Sleep | 31.9 (29.4) | 34.2 (28.4) | 42.9 (31.8) | 49.7 (29.9) | 22.8 (23.9) | 33.2 (28.4) | 33.0 (22.3) | 34.8 (36.4) |
| Stereotypic and motor behaviour | 19.8 (21.7) | 27.8 (22.0) | 45.8 (29.3) | 47.5 (32.0) | 35.3 (32.6) | 50.4 (30.3) | 50.5 (29.1) | 65.2 (25.3) |
| Motivation | 22.6 (22.5) | 36.8 (25.9) | 59.2 (33.4) | 70.4 (28.8) | 33.3 (30.9) | 55.3 (28.8) | 53.2 (31.2) | 64.3 (31.2) |
|  |  |  |  |  |  |  |  |  |
| **ACE-III** |  |  |  |  |  |  |  |  |
| Sample size | 49 | 42 | 82 | 55 | 28 | 16 | 14 | 12 |
| Total | 71.5 (13.1) | 58.2 (16.3) | 75.6 (13.1) | 62.8 (22.7) | 57.1 (13.1) | 42.6 (19.4) | 64.3 (16.3) | 48.4 (23.2) |
| Attention | 77.1 (14.6) | 56.1 (20.0) | 83.1 (14.2) | 66.6 (26.8) | 82.9 (8.1) | 62.9 (23.8) | 86 (9.5) | 71.3 (19.1) |
| Memory | 54.6 (18.6) | 40.2 (17.4) | 69.1 (20.1) | 55.0 (26.9) | 46.1 (17.9) | 35.9 (22.5) | 58.8 (21.5) | 41.7 (26.5) |
| Fluency | 57.0 (21.5) | 43.2 (22.2) | 45.9 (25.1) | 35.3 (28.9) | 30.0 (24.4) | 22.3 (22.3) | 39.8 (27.5) | 26.8 (29.0) |
| Language | 86.5 (12.3) | 77.9 (16.6) | 84.6 (13.8) | 74.5 (23.2) | 45.3 (15.9) | 27.1 (18.8) | 52.7 (20.2) | 38.7 (25.8) |
| Visuospatial | 81.2 (21.1) | 72.1 (25.1) | 89.3 (11.9) | 76.7 (23.5) | 90.9 (13.1) | 76 (24.9) | 90.6 (10.1) | 69.9 (24.6) |
|  |  |  |  |  |  |  |  |  |

Notes. Data presented denote mean values with standard deviations in parentheses. ACE-III = Addenbrooke’s Cognitive Examination third edition; AD = Alzheimer’s disease; bvFTD = behavioural variant frontotemporal dementia; CBI-R= Cambridge Behavioural Inventory Revised; SDL=left predominant semantic dementia; SDR=right predominant semantic dementia.

**Supplementary table 2.** Between-group comparison of behaviour and cognition results at baseline and follow-up

|  | H value^a^ | Post hoc^b^ |
| --- | --- | --- |
| **CBI-R** |  |  |
| Total | 2.337 | - |
| Memory and orientation | 2.513 | - |
| Everyday skills | 2.629 | - |
| Self-care | 12.228** | AD<bvFTD |
| Mood | 1.688 | - |
| Motivation | 1.963 | - |
| Stereotypic behaviour | 5.657 | - |
| Eating habits | 4.845 | - |
| Abnormal behaviour | 1.565 | - |
| Beliefs | 0.799 | - |
| Sleep | 2.368 | - |
| **ACE-III** |  |  |
| Total | 4.958 | - |
| Attention | 2.001 | - |
| Memory | 0.983 | - |
| Fluency | 4.680 | - |
| Language | 24.527** | AD<SDL, AD<SDR, bvFTD<SDL |
| Visuospatial | 1.936 | - |

Notes. ACE-III = Addenbrooke’s Cognitive Examination third edition; AD = Alzheimer’s disease; bvFTD = behavioural variant frontotemporal dementia; CBI-R = Cambridge Behavioural Inventory Revised; SDL=left predominant semantic dementia; SDR=right predominant semantic dementia. ^a^ Kruskal-Wallis tests were used to compare CBI-R and ACE-III changes. ^b^post hoc tests ** denotes statistically significant at Bonferroni adjusted *p* value threshold of 0.01.
